# Neutron Radiography and Tomography of the Drying Process of Screed Samples

**DOI:** 10.3390/jimaging6110118

**Published:** 2020-11-05

**Authors:** Lorenz Kapral, Michael Zawisky, Hartmut Abele

**Affiliations:** TU Wien-Atominstitut, Stadionallee 2, 1020 Wien, Austria; hartmut.abele@tuwien.ac.at

**Keywords:** neutron imaging, screed, Darr method, calcium carbide method, drying behaviour, radiography, tomography, moisture content

## Abstract

The moisture content of screed samples is an essential parameter in the construction industry, since the screed must dry to a certain level of moisture content to be ready for covering. This paper introduces neutron radiography (NR) and neutron tomography (NT) as new, non-destructive techniques for analysing the drying characteristics of screed. Our NR analyses evaluate the results of the established methods while offering much higher spatial resolution of 200 μm, thereby facilitating a two- and three-dimensional understanding of screed’s drying behaviour. Because of NR’s exceptionally high sensitivity regarding the total cross section of hydrogen the precise moisture content of screed samples is obtainable, resulting in new observations. The current methods to measure moisture content comprise the ‘calcium carbide method’, the ‘Darr method’, and electrical sensor systems.

## 1. Introduction

The imaging of building materials has a long tradition at the Atominstitut operating a 250 kW TRIGA reactor. Details of our digitised neutron radiography (NR) instrument can be found in [1] and an overview of our applications in [2]. An essential part of the construction industry is concerned with building materials and their characteristics. In particular, concrete and screed drying properties have often been analysed to optimise construction time. Screed is defined as a layer or strip of material (e.g., concrete, cement) used to level off a horizontal surface such as a floor. The hardening of these materials depends on their moisture content and temperature. If the water content (moisture content) drops below a certain threshold, the screed is ready for covering. The time to reach this specific state varies greatly depending on environmental factors, e.g., humidity, temperature, and properties of the sand, such as particle size, density and affinity for water. [3]. Thus, there is a need for measuring the moisture content of screed samples to optimise time management. Hence, different measuring methods were developed. The most frequently used methods are the ‘calcium carbide method’, the ‘Darr method’ and electrical sensor systems [3,4,5,6,7,8]. Neutron radiography was proven to perform well in detecting hydrogen in various materials [2,9,10,11,12]. The scope of this project is a comparison of neutron radiography with the current methods to show the accuracy of these methods and enable a two-dimensional analysis of the drying behaviour.

As a brief introduction, the ‘calcium carbide method’ (CM) takes advantage of the reaction of calcium carbide with water. According to CM’s protocol, before the measurement, the sample must be shredded to maximise its surface area. Subsequently, the sample is placed together with a capsule of calcium carbide into a pressure vessel equipped with a barometer. Calcium carbide reacts according to the following chemical reaction: CaC${}_{2}$ + 2H${}_{2}$O ⟶ Ca(OH)${}_{2}$ + C${}_{2}$H${}_{2}$. The resulting gaseous ethin (C${}_{2}$H${}_{2}$) raises the pressure in the vessel and the resulting pressure difference is proportional to the moisture content in the sample. A calibration table converts the measured pressure to a water content mass ratio M% [4].

The Darr method is also called the gravimetric method: At first, a part of the sample is removed and weighed at regular intervals. The next step is to dry the sample at a certain temperature, which was 45 ${}^{\circ }$C in this experiment, to create a calibration point. The ratio of the weights before ($m_{tot}$) and after ($m_{cal}$) the drying process yields moisture content $\psi_{m}$ of the sample at the time of weighing, which is given by Equation (1) [5]:

(1) $$\psi_{m}=\frac{m}{m_{cal}}\times 100=\frac{m_{tot}-m_{cal}}{m_{cal}}\times 100$$

**Figure 1:**
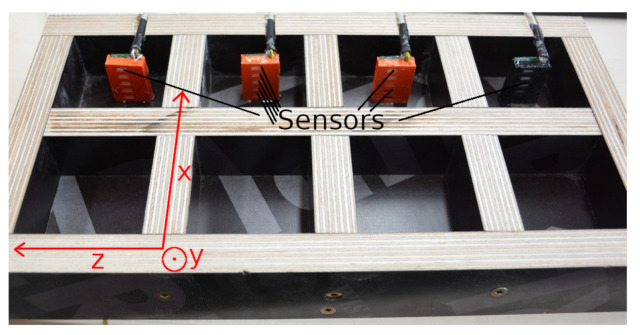
Formwork in which the screed was added. This formwork was used to create eight samples; in four of these samples sensors were placed for electrical measurements. Those samples without electrical sensors were used for destructive methods. 16 h after preparation the samples were removed from the formwork and measured individually. The coordinate system clarifies the orientation of the sensors in the sample relative to the neutron beam, which points in direction of the *z*-axis.

In this experiment, capacity sensors were integrated in the sample at specific heights to create a coarse two-dimensional mapping of the humidity in the sample (shown in Figure 1). These sensors evaluate the water content of the air in the sample by measuring the drop in voltage through the sensor. Water is diamagnetic and, as a result, it changes the diamagnetic properties of the integrated capacitor depending on the humidity in the sample [6,7].

The scintillation detector at Atominstitut of the TU Wien facilitates digitised neutron imaging with a dynamic range of 16 bit and a spatial resolution of 200μm [1,2,13]. The TRIGA reactor can generate a neutron flux up to 2 × 10${}^{5}$ cm${}^{2}$ · s${}^{-1}$ and the affiliated radiography station has a collimation ratio, L/D, of 120. To generate radiography images, a scintillator detector and nitrogen cooled CCD camera, which captured images with a resolution of 512 × 512, are installed; the overall optical pixel resolution amounts to 200 × 200 μm${}^{2}$. After a collection time of five minutes, these images are processed by the analysis tool ‘Image-Pro Plus’ [1,2].

The high scattering cross section of hydrogen results in a high detection sensitivity [14,15]. Moreover, the neutron absorption and scattering in the sand and screed binder is negligible, since the screed consists of materials with very low total neutron cross section e.g., calcium sulfate ($CaSO_{4}$). The intensity of the neutron transmission $I_{tot}$ is then given by [9]:

(2) $$I_{tot}=\Phi_{0}\cdot e^{-\sum_{tot}\cdot d}$$

The neutron transmission is characterised by the thickness of sample *d* and the macroscopic neutron cross section $\sum_{total}=N\sigma =\sum_{s}+\sum_{a}$, where $\sum_{s}$ represents the cross section of the scattered neutrons and $\sum_{a}$ the cross section of the absorbed neutrons. *N* is the number of nuclei per volume, σ is the microscopic cross section [9]. In this experiment, attenuation is mainly attributed to single and multiple scattering as opposed to absorption. If the thickness of the sample is similar to the typical mean path length $L=1/\sum_{total}$, multiple scattering is more likely to occur. The neutron transmission through the sample showed that *L* = 32 cm on average for the used screed samples [15]; in addition, measurements at different sample-to-detector distances showed that multiple scattering is negligible. As a result, the cross section can be approximated by $\Sigma_{i}=\frac{N_{A}\sigma_{i}}{A_{i}}\cdot \rho_{i}$, where $N_{A}$ represents the Avogadro constant and $A_{i}$ the atomic mass [9]. The easiest way to visualise the drying behaviour of screed is by a comparison of the neutron transmissions through the samples at specific time-steps during the drying process, where the majority of the screed’s water evaporates. The drying process causes higher neutron transmission because less water scatters the neutrons. However, a small part remains in the material; therefore, a completely desiccated sample still scatters neutrons, since a part of the water added during the preparation binds with the screed product in a chemical reaction [3]. The previously established methods exclusively detect free water in the samples, i.e., a moisture content of 0 would be measured after heating in an oven, although there is still bound water in the material. To enable comparable results, a calibration point, which indicates the zero point of the established methods, had to be set. The calibration point was selected identically to the Darr method’s calibration point, which means that the samples were dried at 45 ${}^{\circ }$C in a drying oven until they were no longer losing weight by evaporation; in this paper, samples which fulfil this condition are referred as ‘dry’ samples. The NR image of this dry sample, which exclusively contained chemically bound water, was used for the calibration image (index ‘cal’) in Equation (3). To visualise the drying characteristics of screed, the free water which had desorbed during the drying process had to be analysed. Thus, the measured neutron intensity $I_{tot}$ through the sample had to be divided by the intensity of the calibration image $I_{cal}$ to obtain the desired image of the unbound water distribution $I_{free}$ [9].

(3) $$I_{free}=\frac{I_{tot}}{I_{cal}}=\frac{\Phi_{0}\cdot e^{-\sum_{tot}\cdot d}}{\Phi_{0}\cdot e^{-\sum_{cal}\cdot d}}=e^{-\sum_{free}\cdot d}$$

$\Phi_{0}$ is the neutron flux density of the open beam. $\sum_{free}$ represents the cross section of the free water, which desorbed during the drying process; after rewriting $\sum_{free}$, Equation (3) becomes:

(4) $$\ln\left(I_{free}\right)=-\frac{N_{A}\sigma }{A}\cdot \rho \cdot d$$

From Equation (4), the unbound water density of the sample ρ can easily be derived:

(5) $$\rho =-\frac{\ln(I_{free})\cdot A}{N_{A}\sigma \cdot d}$$

Finally, the ratio of the density of water $\rho_{H_{2}O}$ = 1 g/cm${}^{3}$, and the measured density ρ represents the water content of the sample.

(6) $$\Psi_{M}=\frac{\rho }{\rho_{H_{2}O}}$$

## 2. Materials and Methods

Well known concretes and screed types were selected for this experiment [5]. Lafarge EN 197-1 CEM I 52,5 R was prepared according to the data sheet ‘Der Blaue SP’ [16]. This concrete was used for two samples with two different sand-to-binder ratios. In this paper, the sample with the ratio 5:1 will be referred to as ‘CEM I (G/Z = 5/1)’ and the sample with the ratio 6:1 will be called ‘CEM I (G/Z = 6/1)’.

The second material discussed in this paper is ‘Ardex A38 4-h-screed binder’, which was also prepared according to the data sheet’s instructions and will be referred to as ‘A38’ [17]. For all samples, sand of granulation from 5 to 8mm was used.

**Figure 2:**
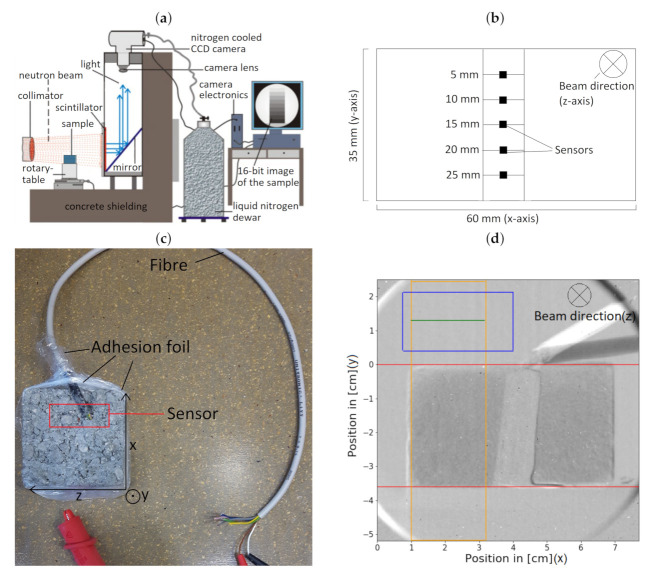
Illustrations of the instrumental setup. (**a**) A collimator directs the neutron beam into the sample, which is placed on a rotary-table. At the scintillator, the transmitting neutrons emit light, which is redirected with a mirror system into a CCD camera. The images are processed by a computer system. (**b**) A schematic of the orientation of the electrical sensors in the sample. (**c**) Wrapping of the sample in adhesion foil to simulate the environment at construction site. The electrical sensorblock, which consists of five sensors, is built in the sample and is connected with a fibre. (**d**) This figure shows an edited image created by the neutron radiography method. An algorithm calculates the averages of the ${\psi_{M}}_{x,y}$ values in the orange box along the horizontal green line according to Equation (7). The blue box represents the ${\psi_{M}}_{x,y}$ values which are used to normalise the images. Values of the open neutron beam are preferred for the normalisation process, therefore the blue box is located above the sample.

To compare all methods, several cuboids with dimensions of 6cm × 6cm × 3.5cm were produced for every sample, since the CM method is destructive. Figure 1 shows the formwork before the screed was added. The electrical sensors were placed in the samples (shown in Figure 1), which were also used for the neutron radiography experiments. Between measurements, all samples were stored in a climatic chamber at a temperature of 22 ${}^{\circ }$C and a relative humidity of 45%RH. To compensate for the small sample size, the samples were wrapped in an adhesion film apart from the top sides; this is shown in Figure 2c. This simulated the environment at construction sites. The adhesion film must be removed shortly before the NR measurements because the foil consists of hydrogen.

Neutron tomography was conducted using the equivalent setup as NR, but a shorter exposure time of two minutes per image was selected. To reduce image artefacts a cylindrical CEM I (G/Z = 6/1) sample with a radius of 3.5 cm and a height of 4 cm was used. The sample is placed on the rotary table between shutter and scintillator. The rotation of the sample is controlled with a stepper motor through a computer program for three-dimensional tomographic measurements. By using the ‘Octopus Reconstruction’ software the filtered back-projection method was applied to create a three-dimensional image, which was coloured with the ‘VG Studio MAX’ software [18,19].

The depth analysis of the drying process is described in Figure 2d. Firstly, the image created by NR must be aligned with the calibration image to calculate the moisture content according to Equation (6). A precise alignment was achieved by location markers on the sample table and a computer algorithm which translated pixels accurately. After this procedure, the image, which can be seen in Figure 2d, shows the water content ${\psi_{M}}_{x,y}$ of the sample at every pixel of the image. To prevent errors created by measuring the neutron transmission through the electrical sensor, which placed in the middle of the sample (shown in Figure 2b), an algorithm was applied. This algorithm calculates the averages of all ${\psi_{M}}_{x,y}$ values in the orange box of Figure 2d along the horizontal green line according to Equation (7). The blue box in Figure 2d represents the ${\psi_{M}}_{x,y}$ values which are used to normalise the images, since the neutron flux of the TRIGA reactor slightly fluctuates.

(7) $${\bar{\psi_{M}}}_{x}=\frac{\sum_{y}{\psi_{M}}_{x,y}}{y}$$

## 3. Results

### 3.1. Neutron Radiography Measurement Results

**Figure 3:**
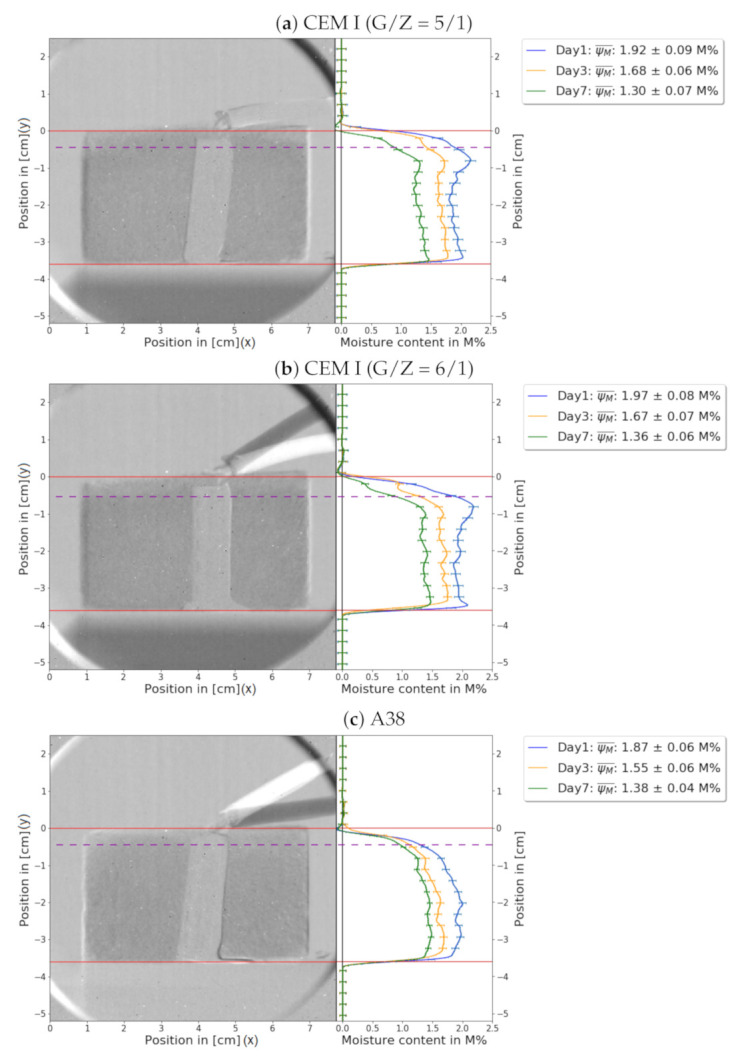
This figure shows the location dependent water content of all samples at 1 day (blue), 3 days (orange), and 7 days (green) after preparation. To compare NR measurements to the established methods, the average water content $\bar{\psi_{M}}$ is shown in the legend. The average water content $\bar{\psi_{M}}$ is calculated according to the equation: $\bar{\psi_{M}}=\sum_{sample}{\bar{\psi_{M}}}_{x}÷x$, where $\sum_{sample}$ involves all indices i in the sample. The orange box in Figure 2d shows the values used to calculate these averages. The red orientation lines indicate the top and the bottom of the sample. The electronic sensor is placed in the middle of the sample. The fibres connecting the sensor to the voltmeter can also be seen on top of the sample. Since the cable was moved between measurements, there is a strong contrast around the fibre. The depth analysis of the drying process of the samples: (**a**) CEM I (G/Z = 5/1). (**b**) CEM I (G/Z = 6/1). (**c**) A38. The measurement shows unexpected results: A thin layer of disproportionate dryness was observed for each sample, which can be seen above the dashed purple line.

The analysis of the drying process depending on the depth is a main goal of this experiment. The drying characteristics of all samples displayed in Figure 3 are fundamentally similar to each other. Due to the adhesion foil, shown in Figure 2c, that is wrapped around the bottom and lateral sides of the sample, evaporation occurred primarily at the top surface. Both CEM I samples show a thin layer of approximately 0.6cm that is significantly dryer than the fairly homogeneously distributed rest of the sample. The difference in diffusion between the water solved in the material and the water in the air would explain this phenomenon. Furthermore, the CEM I samples were slightly wetter than the A38 samples because of a higher amount of water was required for the preparation process.

**Figure 4:**
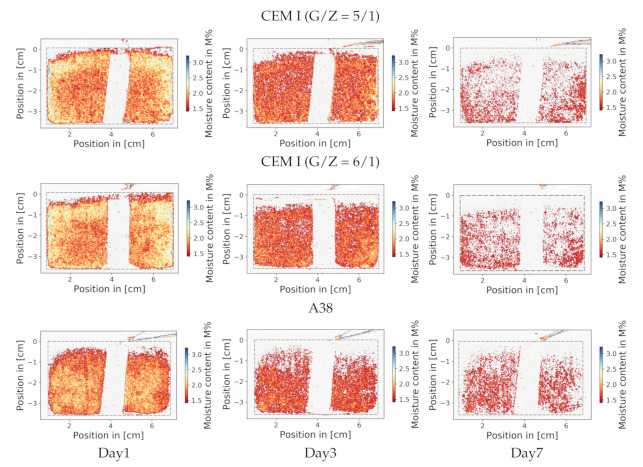
A 3 × 3 matrix of NR images, showing the drying process of 3 different samples over 3 different time-points. As an example, the leftmost column “Day 1” indicates that samples were measured after roughly 24 h. The dashed lines represent the edges of the sample. Blue and yellow colours indicate high water content, and red colours specify low moisture content. To minimise background noises, a water content under 1M% is coloured white. As a result, the sensor and the thin layer of disproportionate dryness also appear in white, which can be seen at the top of the sample.

Figure 4 was created similarly to Figure 3, but instead of greyscale the images were coloured to illustrate the water content. Each pixel represents the moisture content of the sample at its specific location. To reduce the background noise, a filter was applied, so that pixels with low moisture content now appear in white.

Once more, it is illustrated that the upper part of the samples dried faster than the rest. This representation enables an illustration of the structure of the water distribution. One can identify that the water gathers in small grainy microstructures in the space between the sand particles.

### 3.2. Comparison of Neutron Radiography with Established Methods

**Figure 5:**
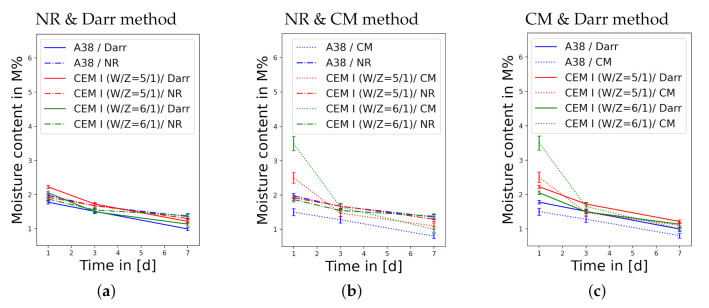
A pairwise comparison of the different methods showing the decrease of moisture content over time; the samples are illustrated in different colours, where the CEM I (G/Z = 5/1) sample is orange, the CEM I (G/Z = 6/1) sample is green and the A38 sample is blue: (**a**) Comparison of NR with the Darr method. (**b**) Distinction between NR and the CM method. (**c**) Comparison of the CM method with the Darr method.

The comparison of neutron radiography with the complimentary methods confirms the reliability and high accuracy of NR. Figure 5 consists of three subplots comparing the established methods with NR.

**Figure 6:**
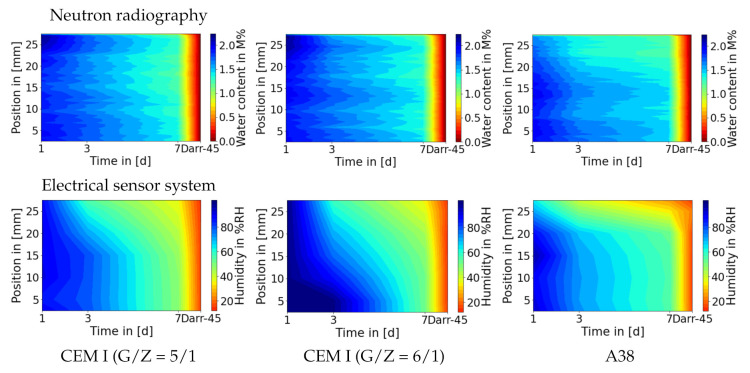
An illustration of the water content over time and location, which resembles the measurements of the electronic sensors which were placed in the sample at different heights. To compensate for the discrepancy in the resolution of the sensors (low, 5 sensors) and NR (high, 200 μm), the measurement results of the humidity sensors were interpolated. In this illustration averages were calculated to create the NR images, therefore the resolution is reduced to 400 μm. Colourised subplots visualise the drying characteristics of the samples over time, where blue equals to high moisture content and red to lower values. After seven days of measuring, the samples were dried in order to create the calibration point. At the location ‘d = 8’ on the time-axis, the tick-marker ‘Darr-45’ represents the water content of the dried calibration sample, which explains the high gradient after day 7. The first row of subplots consists of the results of NR; the second row compares these results to the electrical sensor system’s measurements.

One can see in Figure 5a that the results of the Darr method and NR are consistent, which means that NR confirms the reliability of the Darr method in measuring the water content of concrete or screed samples. However, the comparison with the CM method in Figure 5b shows significant differences, especially at high moisture content values; these discrepancies can also be seen in Figure 5c. The high inaccuracy of the CM method at high water content has been confirmed in the literature [4,5]. Due to the technical possibility to measure the moisture content at any location in the sample with NR, the water content is illustrated over time and location in Figure 6.

The comparison in Figure 6 shows the previously discussed phenomena again. There is a thin layer on the top of the screed which dries disproportionately faster; the subplots regarding the A38 sample confirm this behaviour. The dry layer on the top of the CEM I is not detected by the sensors, because the highest sensor is located under this layer. In Figure 3, one can see that the dry layer in the A38 sample is thicker; both methods show a similar thickness of these disproportionate dry layers. Moreover, the reliability of NR is also confirmed by the electrical sensor systems.

### 3.3. Neutron Tomography

A completely dried CEM I (G/Z = 6/1) was selected for the NT measurements, since the moisture content of a wet sample would change during the measuring process. The three-dimensional visualization was reconstructed out of 251 images of the sample; thereby the sample was rotated for 0.72${}^{\circ }$ between each measurement. Although the sample’s dryness is close to the calibration point, water is highlighted; this water is chemically absorbed.

**Figure 7:**
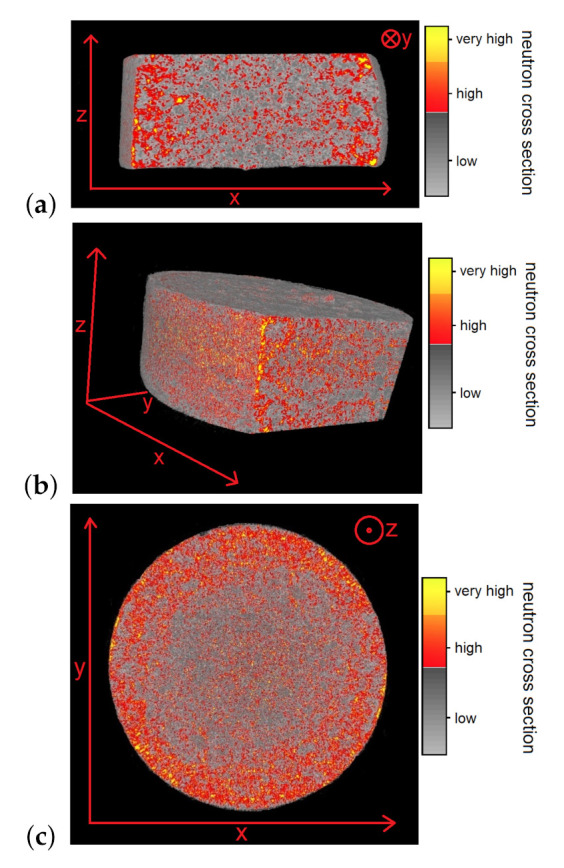
This figure shows the three-dimensional density distribution of a cylindrical CEM I (G/Z = 6/1) sample, which contains only residual water, created by neutron tomography. Different macroscopic neutron cross sections of the material are differently coloured, where the highest cross sections are illustrated in yellow, high cross sections in red and low values in greyscale. The (**a**) A Vertical section through the sample; a Gaussian filter was used to reduce the noise. (**b**) A vertical section through the sample in a different viewing angle. (**c**) A horizontal section through the sample.

The illustrations in Figure 7 show that materials with higher macroscopic neutron cross sections are more likely to be located near surface area: The red dots are pores in which water can be found and can be seen to be more frequent in the periphery of the sample. Moreover, it can be observed that water gathers in small grainy microstructures, which is consistent to the results of NR (Figure 4).

## 4. Discussion

The comparison of neutron radiography with the established methods confirms that NR is superior due to its much better resolution, sensitivity, and non-destructive character in detecting hydrogen in various materials. In addition, the Darr method and the electrical sensor system have been proven as a reliable method to measure the water content in concrete or screed samples. However, the CM method showed high inaccuracy at high levels of moisture content. NR and NT facilitate the technical possibility of two- and three-dimensional analysis of the drying process, which contribute to a better understanding of the drying characteristics. A thin layer of disproportionate dryness was observed at the top of the samples. The two- and three-dimensional illustrations show that water gathers in small grainy microstructures.

## Abbreviations

The following abbreviations are used in this manuscript:

NR	Neutron radiography
NT	Neutron tomography
CEM I (G/Z = 5/1)	Lafarge EN 197-1 CEM I 52,5 R with sand-to-binder ratio of 5:1
CEM I (G/Z = 6/1)	Lafarge EN 197-1 CEM I 52,5 R with sand-to-binder ratio of 6:1
A38	Ardex A38 4-hour-screed binder
CM	Calcium carbide method
RH	Relative humidity
